# The Challenges and Promise of Complement Therapeutics for Ocular Diseases

**DOI:** 10.3389/fimmu.2019.01007

**Published:** 2019-05-15

**Authors:** Dong Ho Park, Kip M. Connor, John D. Lambris

**Affiliations:** ^1^Department of Ophthalmology, School of Medicine, Kyungpook National University, Kyungpook National University Hospital, Daegu, South Korea; ^2^Angiogenesis Laboratory, Department of Ophthalmology, Massachusetts Eye & Ear Infirmary, Boston, MA, United States; ^3^Department of Ophthalmology, Harvard Medical School, Boston, MA, United States; ^4^Department of Pathology and Laboratory Medicine, Perelman School of Medicine, University of Pennsylvania, Stellar Chance Laboratories, Philadelphia, PA, United States

**Keywords:** age-related macular degeneration, complement system, immune modulation, ocular inflammation, therapeutics

## Abstract

Ocular inflammation is a defining feature of sight threating diseases and its dysregulation can catalyze and or propagate ocular neurodegenerative maladies such as age-related macular degeneration (AMD). The complement system, an intrinsic component of the innate immunity, has an integral role in maintaining immune-surveillance and homeostasis in the ocular microenvironment; however, overstimulation can drive ocular inflammatory diseases. The mechanism for complement disease propagation in AMD is not fully understood, although there is accumulating evidence showing that targeted modulation of complement-specific proteins has the potential to become a viable therapeutic approach. To date, a major focus of complement therapeutics has been on targeting the alternative complement system in AMD. Recent studies have outlined potential complement cascade inhibitors that might mitigate AMD disease progression. First-in-class complement inhibitors target the modulation of complement proteins C3, C5, factor B, factor D, and properdin. Herein, we will summarize ocular inflammation in the context of AMD disease progression, current clinical outcomes and complications of complement-mediated therapeutics. Given the need for additional therapeutic approaches for ocular inflammatory diseases, targeted complement modulation has emerged as a leading candidate for eliminating inflammation-driven ocular maladies.

Age-related macular degeneration (AMD) is the leading cause of blindness of the elderly in the Western world (1). Its prevalence is expected to rise as aging populations rise and ~288 million people will be affected by AMD by 2040 (2). The buildup of debris within retinal pigment epithelial (RPE) cells and under the RPE layer is considered a hallmark of AMD development and progression (3). Photoreceptors require a large membrane surface area for phototransduction, and have a large outer segment that must be continuously replaced. The RPE are specialized cells that phagocytize the shed outer segments of photoreceptors, and are the most active phagocytizing cells of the body (4). As aging progresses, it is thought that the machinery of these cells deteriorates, along with their capacity to degrade and recycle photoreceptor metabolic waste. During the lifetime of a person, the choroidal capillary area becomes thinner, and Bruch's membrane may also accumulate lipoprotein material from the choroidal capillaries, which adds to the incompletely digested material deposited by RPE cells. Collectively, this accumulating material is referred to as drusen, which is a major pathological feature of AMD.

Drusen size can predict the development of AMD. Accordingly, a new clinical classification scheme for AMD using drusen size and presence of pigmentary abnormalities has been introduced by the Beckman Initiative for Macular Research Classification Committee (5). In this scheme, individuals over 55 years of age with small (< 63 μm) drusen were considered to have normal aging. Early AMD is characterized by the presence of medium-sized drusen (≥63 and < 125 μm) with no pigmentary abnormalities, and is accompanied by a mild loss of vision. Intermediate AMD is characterized by larger drusen deposits (≥125 μm) and/or presence of pigmentary abnormalities, and is associated with a moderate loss of vision. Late/Advanced AMD is characterized by the presence of any choroidal neovascularization (CNV), known as neovascular AMD, or gross pigment abnormalities and cellular degeneration such as geographic atrophy (GA). Collectively, advanced AMD causes severe loss of vision and can lead to blindness. The incidence of late-stage AMD increases exponentially with age, with the two forms of late AMD, neovascular AMD and GA, occurring with roughly equal prevalence (2, 6). An estimated 1.22 million and 973,000 people in the United States have neovascular AMD and GA in at least one eye, respectively (7). As the population ages, the prevalence of late AMD is expected to rise from 9.6 million to 11.3 million in 2020 and 18.6 million in 2040 (2).

Currently, there is no effective prevention, individual risk estimation, or reliable prognostic evaluation available for the clinical management of AMD. Further, there are no therapeutics approved for the treatment of GA. The development of AMD depends on a complex interplay of risk factors such as age, genetics (8), and behavior including; smoking (9), diet (10), and sunlight exposure (11). Genetic variations in genes involved in the complement system, as well as others, are associated with risk for developing AMD, or risk of progression from early to late AMD (12). Overall, these findings suggest that AMD is a progressive neurodegenerative disease involving inflammation (13), and in particular an inflammatory immune response (14).

## The Complement System is a Vital Component of Innate Immunity

**Figure 1:**
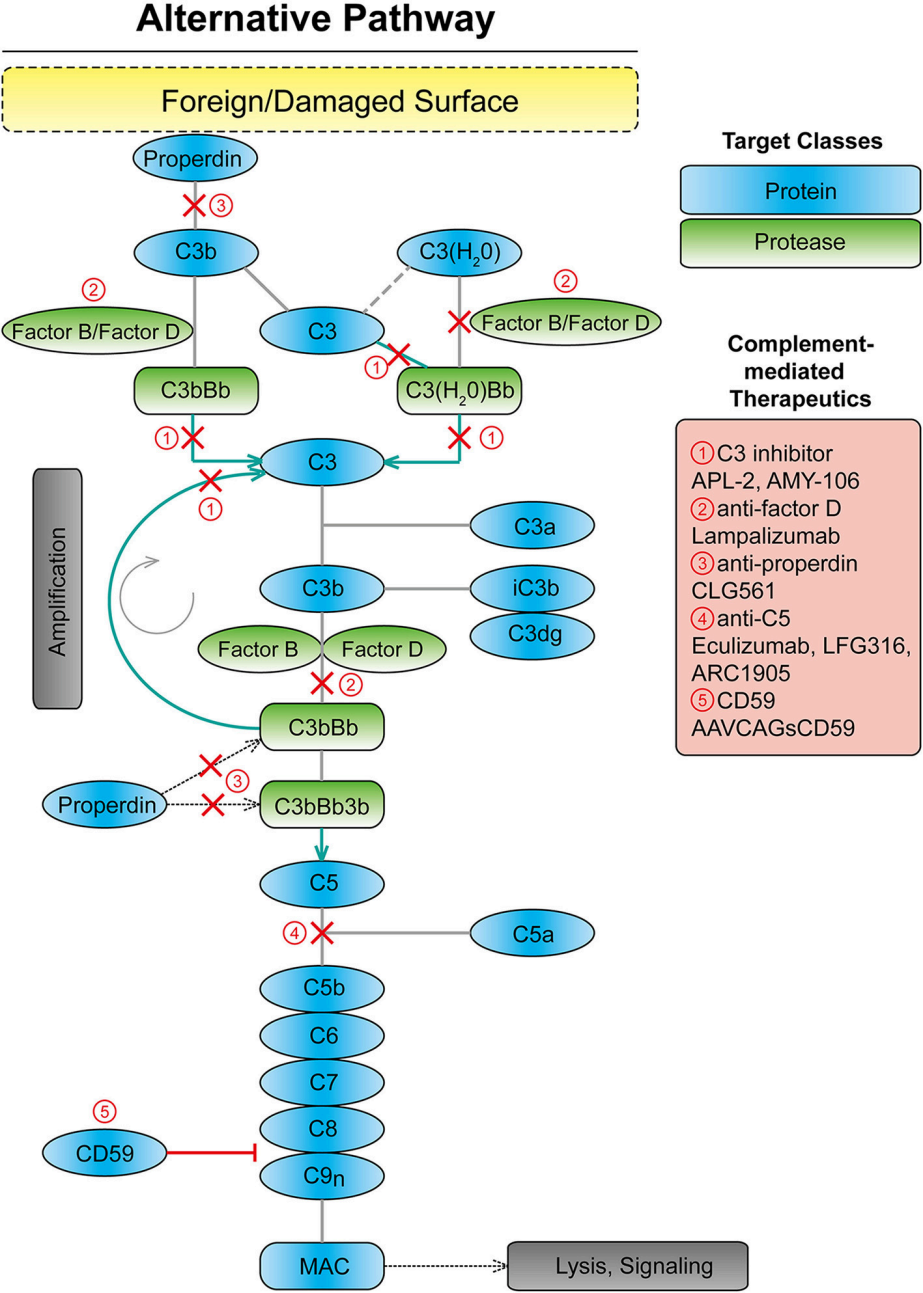
Scheme showing the alternative complement system with targets for complement mediated therapeutics. MAC, membrane attack complex.

The immune system is divided into two distinct types, the innate and adaptive systems. The complement system, as part of the innate immune system, plays an integral role in maintaining immune-surveillance and homeostasis in the ocular microenvironment (15, 16). The complement system consists of three systems classical, lectin, and alternative (17). The classical system is mediated by the binding of the complement component C1q of C1 protein to antigen–antibody complexes. The lectin system is activated by mannose-binding lectin recognition of the polysaccharide or glycoprotein motifs on the cell surface of damaged host and non-host cells (17, 18). Lastly, the alternative complement system is constitutively active through the spontaneous cleavage of an internal C3 thioester bond (17, 19) (Figure 1). The spontaneous hydrolysis of this internal thioester bond within the complement protein C3 forms C3(H_2_O), and while not cleaved, C3(H_2_O) can function in the same manner as C3b in a C3 convertase. This “tick-over” of C3 to C3 (H_2_O) enables it to complex with complement factor B, following by its cleavage by complement factor D, a serine protease, into Ba and Bb fragments. The resulting C3(H_2_O)Bb complex is a C3 convertase, which can efficiently cleave C3 into C3a and C3b. Factor B enables further downstream activation of the alternative system through its binding to C3b (17–21). These components form the C3 convertase enzyme, C3bBb, promoting the cleavage of C3 and creating a positive feedback loop (19, 21). Subsequently, C5 convertase is created by the combination of the C3 convertase with an additional C3b molecule. The C5 convertase then cleaves C5 into C5a and C5b (17–19). C5b recruits C6, − 7, −8, and−9, forming the membrane attack complex (MAC), which forms a pore in the cell membrane causing cell lysis and death, which can be down-regulated by complement inhibitors such as CD59 (22, 23). However, according to a previous immunohistochemical study from human donor eyes with drusen, the antibody against C5b-9 indicating MAC showed a restricted pattern, while the C3 or C5 antibody showed a diffuse pattern throughout the drusen and choroid (24). Thus, it is unclear if MAC formation is linked to AMD disease progression. It may be complement's upstream opsonization functions that are perturbed in this pathology.

## Alternative Complement Cascade Dysfunction in Age-Related Macular Degeneration

### Complement Factor H and Related Proteins

Over a lifetime, the photoreceptors and RPE cells of the retina encounter a number of innate immune activators, such that tight regulation of immunity is necessary to prevent deleterious inflammatory events. Dysregulation of the complement system may potentially drive ocular inflammation, which contributes to vision loss in AMD. Complement byproducts such as complement factor H (FH) in the drusen were the first indication that the complement system is involved in AMD progression (25). In 2005, genome-wide association studies (GWAS) suggested that the complement cascade was involved in AMD disease progression. Table 1 summarizes the genes for the alternative complement pathway that are known to be involved in AMD. Early GWAS and targeted sequencing studies identified a common single nucleotide polymorphism (SNP) in the *CFH* gene (rs1061170) that is associated with an increased risk of developing AMD (26–29). This SNP replaces a tyrosine residue with a histidine residue at position 402 (Y402H) (49). Heterozygous individuals and homozygotes for the Y402H polymorphism have a 2.3 and 5.2-fold increased risk of developing AMD, respectively (30). Approximately 30% of individuals of European descents are carriers of at least one of the Y402H risk alleles (30).

**Table 1:** Genes in the alternative complement pathway that are involved in age-related macular degeneration.

**Complement**	**Gene location**	**Variants**	**Role in complement system**	**References**
Complement factor H (FH)	1q31.3	Y402H (common variant) R1210C, R53C, and D90G (rare variants)	FH is an important regulator of the complement system. Dissociation of the C3Bb complex, known as C3 convertase, is accelerated by FH, thus alterations of FH can lead to abnormally increased complement activity. Furthermore, it acts as a cofactor for factor I.	Common variants (26–30) Rare variants (31–33)
Complement factor H-related (FHR)	1q31.3		Although the exact function is unclear, some of the FHR proteins may act as competitors for FH binding to various ligands and can help to form a novel C3 convertase by binding C3b.	(34–36)
C3	19p13.3	R80G, R102G (common variants) K155Q (rare variants)	C3 is the central component of all three complement systems	Common variants (37–39) Rare variants (40, 41)
Factor B	6p21.33	L9H, R32Q (common variants)	Factor B is cleaved by factor D in the presence of C3b, and its Bb fragment forms the C3bBb, C3 convertase, in the alternative system	Common variants (42–44)
Factor D	19p13.3	rs3826945 (common variants)	Factor D cleaves factor B within this complex into Ba and Bb fragments. This is a rate-limiting step in the formation of the C3bBb complex, which is crucial for the activation of the alternative complement system	Common variants (45)
Factor I	4q25	rs10033900 (common variant) G119R and G188A (rare variants)	This serine protease domain regulates the complement system by cleaving and inactivating C4b and C3b which is regulated by C4bp and FH, respectively	Common variants (41, 46) Rare variants (47)
C9	5p13.1	P167S, R95S (rare variants)	Encodes complement component 9, the final component of the complement cascade and component of the membrane attack complex (C5b-9).	Rare variants (41, 48)

FH is an important regulator of the complement system (50). Increasingly, it has been found that complement activation is linked with AMD associated with genetic variants on chromosome 1 locus (1q32) that contains the *CFH* and *CHFR* genes, rather than chromosome 10 (10q31) around the *ARMS2/HTRA1* genes (51). AMD patients with chromosome 1 mutations have significant complement over-activation in the extracellular matrix of the choriocapillaris, underlying Bruch's membrane. Complement activation and turnover have been identified in the choriocapillaris layer as well, and increased levels are found in individuals with AMD (52, 53). This is also found in donor eyes from individuals genetically at risk for AMD, but who do not yet have clinical manifestations of the disease (54). The activation of the alternative complement pathway is initiated by the formation of the C3bBb complex, also known as C3 convertase. Formation of this complex leads to the amplification of complement signaling and immune response. FH accelerates dissociation of this C3bBb complex, inhibiting alternative complement system activation. Furthermore, FH, as a cofactor, facilitates factor I-mediated C3b inactivation. FH is anchored to the extracellular matrix and the cell surface through interactions with glycosaminoglycans (GAGs) (55). The Y402H polymorphism does not alter the overall protein structure (56). However, the Y402H polymorphism disrupts binding of the complement control region to GAG chains in the Bruch's membrane (55). Because the Y402H variant has decreased binding affinity to numerous components of the damaged retina (57–59), the inhibitory effect of FH on the complement system is thought to be decreased. This could result in poorly controlled complement turnover and excessive chronic local inflammation. The FH and factor H-like protein (FHL-1), components of the alternative system, are capable of suppressing complement activation on the extracellular matrix (51). The shortened splice variant of FH and FHL-1 appears to prevail in the ECM in/around Bruch's membrane (60–63). Because FHL-1 only has the one GAG-mediated anchoring site in its CCP7 domain which anchors FHL-1 to Bruch's membrane and the intercapillary septa, this GAG-binding site is affected by the Y402H polymorphism. In contrast, FH has two anchoring sites and is not particularly affected by the Y402H polymorphism (51). This may explain why the Y402H polymorphism has a disproportionate effect on protein anchoring via GAGs.

Other rare variants in *CFH* SNPs have also been reported to affect AMD, including the R1210C, R53C and D90G polymorphisms (31, 32). R1210C is extremely rare, with a minor allele frequency of 0.0173 %, but has an even stronger association with AMD than Y402H, potentially by acting as a functionally null allele (31). The highly penetrant R1210C variant is associated with a 6-year earlier onset of AMD with drusen phenotype (33). Advanced age and decreased FH induced sub-RPE deposit formation leading to complement activation, which contributed to RPE damage and visual function impairment (64).

A number of other SNPs further downstream on chromosome 1 are associated with AMD, suggesting involvement of the five factor H-related (FHR) proteins in disease pathogenesis (34). Although the exact function of FHR proteins is unclear at present, some of them may bind competitively to ligands involved in FH binding and may facilitate formation of a novel C3 convertase by binding C3b (65). Furthermore, a haplotype of complete deletion of the genes for *FHR-1* and *FHR-3* was associated with a decreased risk of AMD in human studies (34–36).

### C3

C3 is the central component of all three complement systems, and functional changes in C3 directly affect the downstream cascade (66). A common SNP, rs2230199 (R80G), is associated with increased risk of AMD (odds ratio 2.6) (37, 38). The R102G polymorphism of *C3* results in reduced FH binding to the 102G variant. As FH acts as a cofactor for factor I-mediated cleavage of C3b, R102G polymorphism of *C3* decreased the factor I-mediated cofactor activity. By extending the convertase lifetime, activation of the alternative complement system is enhanced (39). A single rare variant in *C3*, K155Q, is positively associated with AMD risk (odds ratio 3.8) (41). K155 in *C3* is in close proximity to the FH binding site (40). In surface plasmon resonance experiments, binding of this *C3* mutant to FH was reduced compared to wild type *C3*. The K155Q allele in *C3* results in resistance to proteolytic inactivation by FH and CFI. Taken together, these findings implicate that loss of C3 functionality contributes to AMD pathogenesis (41). Increased plasma complement protein is also associated with advanced stages of AMD, further supporting the hypothesis that complement activation may be correlated with disease progression as well (67, 68). C3a was significantly increased in AMD patients, ranging from 4.6 to 87.2 % (69). Furthermore, C3a and C5a are markers of acute activation of the alternative complement system. Thus, the plasma concentrations of activation peptides including C3a and C5a are positively correlated biomarkers of AMD (69).

### Factor B

Both haplotypes, L9H variant of *factor B* and the E318D variant in *C2*, as well as a variant in intron 10 of *C2* and the R32Q variant of *factor B*, are considered highly protective against AMD development (odds ratio 0.45 and 0.36, respectively), although the protection is likely mediated by *factor B* mutations (42, 43). Factor B fragments are found in the drusen at similar levels to that of factor H, and convertase formation, which strengthens complement activation, is decreased by the R32Q mutation (44). Furthermore, studies in mouse models of complement dysregulation support a causative role for the complement cascade in multiple retinal pathologies. Factor B is up-regulated in the retinal detachment mouse model, and in the vitreous from patients with retinal detachment (70). In addition, *factor B* knockout mice are protected from retinal detachment associated photoreceptor death which further emphasizes a role for this system in photoreceptor degeneration.

### Factor D

Factor D is one of the serine proteases that regulates the activation of the alternative complement system (71). Factor D becomes transiently active and is able to cleave factor B within this complex into Ba and Bb fragments. This is a rate limiting step in the formation of C3bBb which strengthens the signal and consequently activates the complement system (21, 71). A small case-control series showed that *factor D* gene SNP rs3826945 is positively associated with AMD risk (odds ratio 1.44) (45).

### Factor I

The *factor I* gene spans 63 kb and contains 13 exons, the first 8 of which encode the heavy chain and the last 5 the light chain, which contains a serine protease domain (72). The serine protease domain is responsible for cleaving and inactivating C4b and C3b (73). Factor I-mediated C3b inactivation is assisted by FH. The FH acts as a cofactor for factor I-mediated cleavage of C3b (74), and thus accelerating breakdown of the alternative system C3 convertase, C3bBb. A common polymorphism near the *factor I* gene, rs10033900, has been described (46). A cohort study of 2,493 advanced AMD cases and controls showed that 7.8% of AMD cases compared to 2.3% of controls are carriers of rare missense *factor I* variants (odds ratio 3.6) (41). Another cohort study reported that two variants of *factor I*, G119R and G188A remained significant (47). The rare *factor I* G119R variant conferred a particularly high risk of AMD (odds ratio 22.2). Functional studies on the G119R variant showed that the secretion of the mutant protein was lower than that of the wild type protein, which led to a decrease in factor I-mediated cleavage of C3b.

### Complement 9

Complement component 9 (C9) is the most downstream component of the terminal complement complex cascade, acting as the terminal effector molecule of all three complement activation systems. Because the ultrastructure terminal complement complex is partially dependent on the amount of C9 molecules relative to C5b-8 molecules, a significant reduction in C9 can alter the terminal complement complex stoichiometry, reducing cytolytic activity (75, 76). Genotyping in 5,115 independent samples confirmed associations to AMD with a P167S allele in *C9* (41). When compared to the controls, a two-fold increase was observed for this variant in the AMD cases. Another R95X variant was negatively associated with AMD risk in a small study (48), but has not been confirmed. Together, these findings suggest that the terminal complement pathway has a potential role in AMD pathogenesis.

## Therapeutic Approaches and Clinical Trials Using Complement-Mediated Drugs

In addition to strong genetic and basic research studies supporting a pathological role for the complement system in AMD, many clinical trials for therapeutics targeting alternative complement cascade are at various stages or completed (Table 2). Clinical trials for eight drugs that target the alternative complement cascade include two targeting C3, one targeting factor D, one targeting properdin, three targeting C5, and one targeting CD59.

**Table 2:** Complement-mediated therapeutics in clinical trials for the treatment of age-related macular degeneration.

**Target**	**Drug (Company)**	**Class**	**Phase (Name) trial number**	**Route of administration**	**Indication**	**Design**	**Trial status and primary outcomes**
C3	POT-4/AL78898A (Potentia/Alcon)	Peptide	1 (AsAP) NCT00473928	IVT	nAMD	SAD, *n* = 27	Completed, no safety concerns (77)
			2 (RACE) NCT01157065	IVT	nAMD	Single dose POT-4 (50 μL) vs. ranibizumab (50 μL), *n* = 49	Completed, POT-4 group did not show a reduction in the central subfield retinal thickness from baseline.
			2 NCT01603043	IVT	GA	Monthly POT4 vs. Sham, *n* = 10, 1 year	Terminated
C3	APL-2 (Apellis)	Peptide- PEGylated	1 NCT02461771	IVT	nAMD	SAD	Completed, unpublished
			2 (FILLY) NCT02503332	IVT	GA	Monthly vs. every other month vs. Sham, *n* = 246, 18 months	29% significant reduction in GA growth at 12 months in the monthly injection group
			3 NCT03525613	IVT	GA	Monthly vs. every other month vs. Sham, *n* = 600, 30 months	Recruiting
			1b/2 NCT03465709	IVT	nAMD	Target *n* = 17, 18 months	Recruiting
Factor D	Lampalizumab (Roche)	Fab	1a NCT00973011	IVT	GA	SAD	No safety concerns (78)
			2 (MAHALO) NCT01229215	IVT	GA	Monthly vs. every other month vs. Sham, *n* = 123	There was a trend for the reduction of GA progression by 20% in the monthly group (79)
			3 (CHROMA, SPECTRI) NCT02247479 NCT02247531	IVT	GA	Duplicate trials, *n* = 906 (CHROMA) and *n* = 975 (SPECTRI), every 4 weeks vs. every 6 weeks vs. Sham for 96 weeks	No treatment benefits compared to the sham group in both trials (80)
			2 NCT02288559	IVT	GA	Every 2 weeks vs. every 4 weeks vs. Sham, target *n* = 99, 6 months	Completed, unpublished
Properdin	CLG561 (Novartis)	Monoclonal Ab	2 NCT02515942	IVT	GA	CLG561 vs. CLG561+LFG316 vs. Sham, 12 monthly injections, *n* = 114	Completed, unpublished
C5	Eculizumab (Alexion)	Monoclonal Ab	2 (COMPLETE) NCT00935883	IV	GA, drusen	Low dose (600 mg) weekly for 4 weeks followed by 900 mg every 2 weeks vs. high dose (900 mg) weekly for 4 weeks followed by 1,200 mg every 2 weeks for 24 weeks, then FU 6 months, *n* = 30 GA (COMPLETE)*n* = 30 drusen	Eculizumab was well-tolerated through 6 months but did not decrease the growth rate of GA significantly at 6 or 12 months (81). No reduction of the drusen volume at 6 months (82)
C5	LFG316 (Novartis)	Monoclonal Ab	1 NCT01255462	IVT	GA or nAMD	SAD, *n* = 24	Completed, unpublished
			2 NCT01527500	IVT	GA	Low dose (5 mg/50 μL) vs. Sham, monthly for 1 year, *n* = 150	No reduction of the GA lesion in the treatment group compared to the sham group
			2 NCT01535950	IVT	nAMD	Active vs. Sham, 113 days *n* = 43	Completed, unpublished
			2 NCT01624636	IV	nAMD	Placebo vs. 2 doses of LFG316, 113 days	Terminated, unpublished
C5	ARC1905 (Ophthotech)	Aptamer	1 NCT00950638	IVT	GA	Dose 1 (0.3 mg) vs. Dose 2 (1 mg), *n* = 47, 1 year	Completed, unpublished
			1 NCT00709527	IVT	nAMD	6 monthly ARC1905 (0.3, 1, or 2 mg) in combination with ranibizumab (0.5 mg), *n* = 43, 2 years	Completed, well-tolerated without evidence of acute toxicity (83)
			2b NCT02686658	IVT	GA	Dose 1 vs. Dose 2 vs. Sham, 12 months, *n* = 200	Recruiting
			2a NCT03362190	IVT	nAMD	ARC1905 Dose 1 vs. Dose 2 vs. Dose 3 vs. Dose 4 in combination with ranibizumab 0.5 mg, *n* = 64	Generally well tolerated for 6 months (84)
			2a NCT03374670	IVT	PCV	ARC1905 Dose 1 vs. Dose 2 in combination with aflibercept 2 mg, *n* = 20	Recruiting
			2b NCT03364153	IVT	STGD1	ARC1905 vs. Sham, *n* = 120	Recruiting
CD59	AAVCAGsCD59 (Hemera)	Virus	1 NCT03144999	IVT	GA	SAD (3 dose levels, expansion), *n* = 17	Recruiting

*Ab, antibody; GA, geographic atrophy; IV, intravenous; IVT, intravitreal; nAMD, neovascular age-related macular degeneration; PCV, polypoidal choroidal vasculopathy; SAD, single ascending dose; STGD1, autosomal recessive Stargardt disease 1*.

### C3-Mediated Therapeutics

Inhibiting the C3 protein, including the C3 convertase, may be a desirable therapeutic approach. C3 modulates amplification of all initiation pathways, the generation of anaphylatoxins (C3a, C5a), and the membrane attack complex (MAC), while C3 inhibition attenuates these events. C3 functions as a central hub that mediates and controls the upstream activation and downstream effector functions of the complement cascade. Initial attempts in developing small molecules that inhibit conversion of C3 failed due to lack of potency and specificity (85).

In contrast, the peptidic inhibitor compstatin exerts its function via direct binding to native C3. Compstatin was discovered by screening phage-display libraries searching for C3b-binding peptides (86). Compstatin is a 13 amino acid cyclic peptide that inhibits complement activation by binding C3 and interfering with convertase formation and C3 cleavage (and the subsequent C3b opsonization, amplification, and generation of effectors) (85). Thus, compstatin could be a potent inhibitor, blocking all three complement systems (87).

POT-4 (Potentia Pharmaceuticals, Crestwood, KY), a compstatin derivative, is the first complement-specific drug that has entered clinical trials for the indication of AMD. A phase 1 clinical trial (AsAP, NCT00473928) using intravitreal POT-4 delivery was conducted in patients with neovascular AMD, and suggested that there were no safety concerns (77). Following that, a double-masked, randomized, multicenter phase 2 clinical trial for patients with neovascular AMD (RACE, NCT01157065) was performed. Forty-nine patients were randomized to receive either single POT-4 (50 μL) or single ranibizumab (Lucentis^®^, Roche AG, Basel, Switzerland) (50 μL) injections, and were followed up for 12 weeks. The primary outcome was a mean reduction from baseline in the central subfield retinal thickness at week 4. Compared to the ranibizumab group (199.9 μm), the POT-4 group showed no benefit (-12.1 μm) in the reduction of macular edema (88). For the indication of GA, a phase 2 clinical trial with 10 patients (NCT01603043) was terminated before completion, and product deposits was reported in four cases among seven patients (57.4%).

The observation of product precipitation, led to an attempt to increase its solubility by pegylation of POT-4 with a 40,000 Da PEG (termed APL-2, Apellis Pharmaceuticals Inc., Crestwood, KY); APL-2 is currently under clinical trials for patients with GA and neovascular AMD. A phase 2 clinical trial (FILLY, NCT02503332) evaluated the efficacy of APL-2 by comparing three groups: a monthly intravitreal APL-2 injection (15 mg/0.1 mL) group, every other month APL-2 injection (15 mg/0.1 mL) group, and the sham group. The monthly intravitreal APL-2 injection group (*n* = 84) showed a 29% significant reduction in GA growth at 12 months compared to the sham group (*n* = 80) (89). The every other month APL-2 injection group (*n* = 78) showed a 20% reduction in GA growth, though this decrease was not statistically significant. Best-corrected visual acuity (BCVA) did not differ among the three groups. Of concern was that individuals receiving APL-2 had an 8 and 18% conversion rate to neovascular AMD in the every other month and monthly injection groups, respectively (90). Conversion to neovascular AMD appeared to be dose dependent based on monthly and bi-monthly injections. A possible explanation is that PEG can induce CNV in a mouse model by upregulating complement proteins in the RPE-choroid tissue or that a complement inhibition-mediated shift of pro-inflammatory M1 to pro-angiogenic M2 macrophages as part of the repair process, may lead to small exudations (91, 92). Endophthalmitis occurred in two out of 86 patients (1.3%) and in one out of 79 patients (2.3%) in the every other month and monthly injection groups, respectively (93). At present, a new 30-month, Phase 3, multicenter clinical trial for GA is recruiting subjects (NCT03525613) (94). Patients will be randomized to monthly and every other month injections for both APL-2 (15 mg/0.1 mL) and sham groups. Additionally, a phase 2 clinical trial for patients with neovascular AMD has been initiated, and is currently recruiting subjects (NCT03465709) (95).

In the meantime, a novel non-pegylated C3 inhibitor is under development (termed AMY-106, Amyndas Pharmaceuticals) and has shown prolonged residence in ocular tissues at C3-saturating levels, extending over 3 months after a single intravitreal injection in cynomolgus monkeys (96). The increased bioavailability of this C3 inhibitor, indicates its clinical potential for ocular indications associated with C3 convertase dysregulation (e.g., AMD).

### Factor D-Mediated Therapeutics

#### FCFD4514S (Lampalizumab)

As a rate limiting enzyme, factor D cleaves factor B to generate C3 convertase and is a pivotal activator of the alternative complement system (21, 97). FCFD4514S (lampalizumab, Roche AG, Basel, Switzerland), a Fab fragment of humanized IgG murine anti-factor D antibody, was developed to block factor D-mediated formation of the C3 convertase (98). A phase 1 trial (NCT00973011) of intravitreal lampalizumab injection in patients with GA found that this modality is safe and well-tolerated, and there were no adverse events (78). This was followed by the phase 2 MAHALO study. “The phase 2 clinical trial (MAHALO, NCT01229215) was a multicenter, randomized, controlled study that evaluated intravitreal delivery of lampalizumab (10 mg/100 μL) administered monthly (*n* = 42) and every other month (*n* = 41) vs. sham control (*n* = 40) in patients with GA. The primary endpoint was the mean change in the lesion area from baseline to month 18 as measured by fundus autofluorescence (79).” The results showed a trend for a reduction in GA progression of 20% in the lampalizumab group, but there was no significant difference between the lampalizumab and sham groups (79). Interestingly, in a subgroup of *factor I* risk-allele carriers (57% of the patients), monthly treatment showed a 44% reduction in GA area progression compared to the sham control (95% CI, 15 to 73%). “The MAHALO study demonstrated an acceptable safety profile during the 18-month treatment period. The MAHALO study concluded that modulation of the complement system can affect the progression of GA, which is supported by human genetic studies for which various genetic variants in the alternative complement system increase the AMD risk (79).”

However, the subsequent following phase 3 trials showed conflicting results. Two similarly designed phase 3 double-masked, randomized, sham-controlled clinical trials (CHROMA, NCT02247479 and SPECTRI, NCT02247531) enrolled participants at 275 sites in 23 countries (99, 100). “Participants were aged 50 years or older, with bilateral GA and no prior or active CNV in either eye. Participants were randomized 2:1:2:1 to receive 10 mg of intravitreal lampalizumab every 4 weeks, a sham procedure every 4 weeks, 10 mg of lampalizumab every 6 weeks, or a sham procedure every 6 weeks, through 96 weeks. The primary endpoint was the mean change in the GA area at 48 weeks by fundus autofluorescence (80).” In contrast to the favorable results from the phase 2 study, the phase 3 study CHROMA (*n* = 906) showed both dose arms of the monthly and every 6-week injections did not show any significant efficacy compared to the sham group (80). The SPECTRI study (*n* = 975) also demonstrated no treatment benefit of lampalizumab. No benefit of lampalizumab was observed across prespecified subgroups, including by factor I, noted in the earlier phase 2 study. It was noted that development of new CNV in patients with bilateral GA occurred in < 2% of the study or fellow eyes. This finding is consistent with observational studies, which reported conversion rates of 2% at 2 years and 11% at 4 years in patients with bilateral GA and no baseline CNV (101). CHROMA and SPECTRI, which are the largest GA complement studies conducted to date, concluded that lampalizumab did not reduce GA enlargement vs. sham over 48 weeks of treatment. The limitation of these studies comes from the selection criteria of enrolled patients which excluded patients with smaller or larger lesions, unilateral GA, autofluorescence patterns except for banded or diffuse, current or prior CNV, GA from causes other than AMD, or earlier stages. Recent diffusion studies with enriched Bruch's membrane from human donor eyes over a wide age-range demonstrated that there is distinct selectivity in the permeability of Bruch's membrane to complement proteins with size and glycosylation being the important determinants (102). Factor D can penetrate Bruch's membrane suggesting a possible reason as to why the clinical trials with lampalizumab may have failed. Furthermore, an *in vivo* study with mice reported that the absence of both factor H and factor D pushes the complement system identifying a factor D bypass mechanism that is likely always present but only clinically germane in association with factor H dysfunction (103).

### Properdin-Mediated Therapeutics

C3 convertase (C3bBb) is unstable, and is stabilized by the binding of properdin. Stabilized C3bBb can cleave more C3, thus generating a feedback loop (17, 19). Therefore, it is hypothesized that use of an anti-properdin antibody should destabilize the C3 convertase and block the feedback loop. An anti-properdin monoclonal antibody (CLG561, Novartis International AG, Basel, Switzerland) was developed by Novartis, and a phase 2 clinical trial (NCT02515942) was completed. The purpose of that study was to evaluate the safety and efficacy of 12 (every 28 days) intravitreal injections of CLG561 as a monotherapy and as a combination therapy with LFG316 (anti-C5, discussed below) compared to the sham in subjects with GA. However, to date the results remain unpublished (104).

### C5-Mediated Therapeutics

Cleavage of C5 generates C5a and the initiation of MAC, which are key mediators of the terminal complement pathway and complement activation (18). C5a is immunomodulatory, while the MAC initiates cell lysis. Histopathologic specimens of human dry AMD lesions strongly stain for C5 and MAC at the key sites of pathology (24). C5 inhibitors do not affect the formation of upstream complement components such as C3b, which are important in host defense mechanisms. It was suggested that by inhibiting C5-mediated inflammatory and MAC activities, a therapeutic benefit may be achieved in both dry and wet AMD while sparing the immunoprotective functions of the complement system, however, this has not yet been proven in the clinic (105). Three anti-C5 antibodies are in clinical trials: eculizumab (Soliris^®^; Alexion Pharmaceuticals, Cheshire, CT, US), LFG316 (Novartis Pharma AG, Basel, Switzerland), and ARC1905 (Zimura^®^, Ophthotech Corp., Princeton, NJ, US).

#### Eculizumab

Eculizumab is a humanized monoclonal antibody derived from the murine anti-human C5 antibody m5G1. This antibody specifically binds the terminal complement protein C5, thereby inhibiting cleavage to C5a and C5b during complement activation and preventing MAC formation. The United States Food and Drug Administration (FDA) and European Medicines Agency (EMA) approved eculizumab for the treatment of two rare genetic deficiencies of complement inhibition, atypical hemolytic uremic syndrome and paroxysmal nocturnal hemoglobinuria (106). Recently, the FDA/EMA approved eculizumab for the treatment of adult patients with generalized myasthenia gravis, who are anti-acetyl-choline receptor antibody positive (107).

A phase 2 clinical trial (COMPLETE, NCT00935883) was the first prospective, randomized, placebo-controlled investigation of complement inhibition for the treatment of AMD (108). This trial was conducted to evaluate the effect of intravenous injection of eculizumab, a systemic C5 inhibitor, on the expansion of GA in patients with AMD (81). “Patients were randomized 2:1 to receive intravenous eculizumab or placebo over 6 months. In the eculizumab treatment arm, the first 10 patients received a low-dose regimen of 600 mg weekly for 4 weeks followed by 900 mg every 2 weeks until week 24, and the next 10 patients received a high-dose regimen of 900 mg weekly for 4 weeks followed by 1200 mg every 2 weeks until week 24. At 26 weeks, the GA area increased by 0.19 ± 0.12 and 0.18 ± 0.15 mm in the eculizumab and placebo groups, respectively (*P* = 0.96). At 52 weeks, the GA area increased by 0.37 ± 0.22 mm in the eculizumab group and by 0.37 ± 0.21 mm in the placebo group (*P* = 0.93). None of the eyes converted to neovascular AMD (81).” The lack of any trend toward therapeutic efficacy with eculizumab led the study authors to conclude that eculizumab was well-tolerated through 6 months but did not decrease the growth rate of GA significantly. Furthermore, the effect of eculizumab treatment on the drusen volume was also evaluated (82). The mean drusen cube root volumes were 0.49 and 0.47 mm (*P* = 0.64) at baseline and 0.51 and 0.42 mm (*P* = 0.17) at 26 weeks in the eculizumab and placebo groups, respectively, which showed no beneficial effect of eculizumab in reducing the drusen volume compared to the placebo.

There are several limitations in the COMPLETE clinical trial, including the route of administration. The rationale for using a systemic drug in that clinical trial was based on the belief that complement activation in the choroid played an important role in the progression of GA. Furthermore, eculizumab already has been approved for systemic delivery for patients with paroxysmal nocturnal hemoglobinuria (109) and atypical hemolytic uremic syndrome (110); thus, systemic delivery of eculizumab was possible without additional safety studies. Although systemic complement inhibitors are successfully used for other systemic diseases including paroxysmal nocturnal hemoglobinuria, the amount of drug delivered systemically could be inadequate to penetrate the blood retinal barrier and affect the pathological progression of GA. Another possible explanation could be that the clinical trials focused on the wrong targets. Eculizumab for GA is no longer listed among Alexion's pipeline therapeutics (111).

#### LFG316

LFG316 is a human monoclonal C5 antibody. A phase 2 clinical trial (NCT01535950) to evaluate the efficacy of intravitreal LFG316 in patients with neovascular AMD was completed though the results remain unpublished (112). Another phase 2 clinical trial (NCT01527500) for 150 patients with GA was completed. However, the intravitreal LFG316 treatment group (5 mg/50 μL) did not have reduced GA lesions compared to the sham group over 1 year of follow-up (113). A phase 2 clinical trial (NCT01624636) evaluating the safety and tolerability of intravenous LFG316 injection was terminated before completion (114).

#### ARC1905

ARC1905 is an intravitreal anti-C5 aptamer. Results of the phase 1 study (NCT00709527) were presented at the Association for Research in Vision and Ophthalmology 2010 Annual Meeting (83). “Forty-three patients with subfoveal neovascular AMD received six monthly administrations of ARC1905 (0.3, 1, or 2 mg) in combination with ranibizumab (0.5 mg). The BCVA in the study eye was 20/63 to 20/200. It did not show any dose-limiting toxicity during 6 months. The mean change in BCVA at week 24 was an increase of +13.6, +11.7, and +15.3 letters at the doses of 0.3, 1, and 2 mg, respectively. Furthermore, 46%, 47%, and 60% of patients gained 3 or more lines of visual acuity at the doses of 0.3, 1, and 2 mg, respectively. The mean change in center point thickness by optical coherence tomography was−150 μm.” The phase 1 study showed that the combined therapy of C5 and VEGF inhibition was well-tolerated without any toxicity issue. An additional phase 1 trial (NCT00950638), which was conducted to evaluate the safety and tolerability of ARC1905 in patients with GA, was completed though the results have not been published (115).

A phase 2 clinical trial for patients with GA started recruiting (NCT02686658) (116). Furthermore, 64 patients with neovascular AMD were enrolled in the randomized, dose-ranging, open-label, multicenter phase 2a safety trial (NCT03362190) of ARC1905 in combination with ranibizumab (117). Ophthotech announced that after 6 months of treatment, the ARC1905 combination therapy was generally well-tolerated in neovascular AMD. It is interesting that though this clinical trial, with a small sample size, was not designed to detect a significant difference in efficacy, 60% of patients who had received monthly ARC1905 (2 mg) in combination with ranibizumab (0.5 mg) gained greater than or equal to three lines of vision, or 15 Early Treatment of Diabetic Retinopathy Study letters, defined as a significant visual gain (84).

A phase 2a open-label trial to assess the safety of ARC1905 in combination with aflibercept (Eylea^®^, Bayer AG, Leverkusen, Germany) 2 mg, in patients with idiopathic polypoidal choroidal vasculopathy has started recruiting (NCT03374670) (118). Furthermore, a phase 2b randomized, double-masked, controlled trial to evaluate the safety and efficacy of ARC1905 compared to sham in subjects with autosomal recessive Stargardt disease 1 is recruiting (NCT03364153) (119).

#### CD59-Mediated Therapeutics

Soluble and cell-bound regulators of complement including CD59 help to protect healthy host tissue from self-recognition and serve to prevent activation of a complement response (17). However, damaged or diseased host cells can down-regulate membrane-bound complement inhibitors which enables targeted clearance. An imbalance between complement recognition and initiation on healthy host cells can lead to unregulated complement activation, opsonization, and/or subsequent cellular damage. Moreover, accumulation of MAC on cell surfaces leads to cell damage and death, associated with several clinical findings observed in AMD. CD59 functions by binding the C5b678 terminal complement protein complex and preventing the incorporation of the multiple C9 molecules required to complete the formation of a pore in the cell membrane. Normal cells within the human body produce a surface protein, CD59, which blocks formation of the MAC (22, 23). In mouse models with retinal pathologies including retinal detachment (70) and oxygen-induced retinopathy (120, 121), *CD59* expression was suppressed compared to the control mice. The above animal studies showed that the presence of CD59 can have a dichotomous role. *Cd59* down-regulation can either be protective in pathologies involving dividing cells (e.g., vascular endothelial cells in neovascular disease), or can lead to neurodegeneration when *Cd59* down-regulation enables complement to target non-dividing cells of the central nervous system (e.g., photoreceptor cell). In AMD, the complement cascade is thought to be upregulated and it has been postulated that targeting MAC formation can protect against self-cell death. AAVCAGsCD59 (Hemera, QC, Canada), an ocular gene therapy product that is delivered intravitreally, causes normal retinal cells to increase the expression of a soluble form of CD59 (sCD59). This soluble recombinant CD59 is designed to protect retinal cells by inhibiting MAC formation. Adeno-associated virus (AAV) serotype 2 was used because it has been shown to be safe for use in humans and is generally considered less immunogenic than adenovirus vectors (122). A phase 1 trial (NCT03144999) was initiated to evaluate drug safety after a single injection of AAVCAGsCD59 administered in an office setting for patients with GA (123). Regarding the frequency of injections, the biggest advantage of this gene therapeutic approach is that it requires only one injection compared to other drugs with monthly or bimonthly intravitreal injections. This could be beneficial in minimizing potential endophthalmitis, which is a risk that can arise from multiple injections.

## Treatment Challenges

Vascular endothelial growth factor (VEGF) drives ocular neovascularization, and anti-VEGF therapies are highly effective in the treatment of neovascular AMD. Although these drugs are highly effective, they require frequent intraocular injections, and are costly, reducing patient compliance. Furthermore, anti-VEGF therapies are not effective in treating GA.

A significant body of work in animal models, genetic studies and clinical trials suggests an important but complex role for the complement system in AMD, including GA. However, therapies targeting the alternative complement cascade have thus far had only modest therapeutic effects in GA and neovascular AMD. The reasons for these unexpected outcomes have not been fully elucidated, but are likely due the disease stages treated, and in some cases, insufficient drug delivery. The complement system may be more relevant in the earlier stages of the disease, before clinical pathologies such as GA, CNV and decreased visual acuity develop. Drug delivery may also have been problematic in some studies such as intravenous eculizumab injection in the COMPLETE study. Intravitreal injections may be the most popular, but there are issues with continued dosing and getting to the right locations within the eye (i.e., monthly injections). Gene therapy has made great strides recently, and sub-retinal injections of AAV-delivered therapeutics are now FDA approved for other ocular indications such as inherited retinal dystrophy (i.e., Voretigene neparvovec, Luxterna, Spark Therapeutics Inc.) (124), which could show the beneficial mode of delivery of the future complement therapeutics. In addition, an emerging hypothesis points toward a dual role for complement in the progression of age-related and degenerative diseases, which are often driven by accumulating debris (125). However, a previous study using a primate model with early-onset macular degeneration, which develop drusen in < 2 years after birth, showed that intravitreal C3 inhibitor compstatin injection for 6 months resulted in drusen disappearance (126). The “fitness” of the cascade, largely defined by the complotype of polymorphisms/mutations in complement genes (127), is likely of high importance in these chronic, slowly developing disorders.

## Conclusion

Abundant evidence from pathological as well as genetic studies has contributed to a breakthrough in our understanding of the role of the complement system in the pathogenesis of AMD. Thus, local inhibition of complement activation has been considered a promising approach for treating both forms of late AMD. In light of the probable role of the complement system in development of AMD, many clinical trials investigating the effect of complement inhibitors have been conducted or are in progress. The results of clinical trials, in which often only a subgroup of patients responded favorably, has shown that careful stratification of indications and patient cohorts will be critical to identify patients that may benefit from complement-mediated therapies. In this aspect, genetic and/or clinical diagnostic tools will be important. Continued research, including studies on the initial development of late AMD and the subsequent impairment of visual function, will be crucial to further understand the complement pathophysiology in AMD, and to identify additional potential therapeutic targets for complement modulation. Furthermore, the conceptual diversity of complement-mediated therapeutics could allow for accessible indications, treatment options, costs, and clinical availability. Thus, the results of ongoing clinical trials are eagerly awaited, with the hope of developing additional therapeutic modalities for this increasingly common malady.

## Author Contributions

All authors listed have made a substantial, direct and intellectual contribution to the work, and approved it for publication.

### Conflict of Interest Statement

JL declares that he is the founder of Amyndas Pharmaceuticals, is named as an inventor on patents or patent applications describing the therapeutic use of complement inhibitors (some of which are being developed by Amyndas Pharmaceuticals) and is the inventor of the compstatin analog licensed to Apellis Pharmaceuticals termed 4(1MeW)7 W (also known as POT-4 and APL-1) and pegylated derivatives such as APL-2. The remaining authors declare that the research was conducted in the absence of any commercial or financial relationships that could be construed as a potential conflict of interest.
